# Paying for prevention in clinical practice: Aligning provider remuneration with system objectives

**DOI:** 10.1186/1472-6831-15-S1-S7

**Published:** 2015-09-15

**Authors:** Stephen Birch

**Affiliations:** 1 Centre for Health Economics and Policy Analysis, McMaster University, Canada; 2 Manchester Centre for Health Economics, University of Manchester, UK

## Abstract

Evidence on the efficacy of preventive procedures in oral health care has not been matched by uptake of prevention in clinical practice. Reducing oral disease in the population reduces the size of the future market for treatment. Hence a provider's intention to adopt prevention in clinical practice may be offset by the financial implications of such behaviour. Effective prevention may therefore depend upon prevention-friendly methods of remuneration if providers are to be rewarded appropriately for doing what the system expects them to do. This paper considers whether changing the way providers are paid for delivering care can be expected to change the utilisation of preventive care in the population in terms of the proportion of the population receiving preventive care, the distribution of preventive care in the population and the pattern of preventive care received. A conceptual framework is presented that identifies the determinants of rewards under different approaches to provider remuneration. The framework is applied to develop recommendations for paying for prevention in clinical practice. Literature on provider payment in dental care is reviewed to assess the evidence base for the effects of changing payment methods, identify gaps in the evidence-base and inform the design of future research on dental remuneration.

## Background

Substantial evidence exists about the efficacy of preventive procedures in oral health care. However data from surveys of oral health in populations indicate that considerable levels of oral disease, both untreated and treated, still occur [1,2] causing reductions in health-related well-being of the individual, through pain, suffering and reductions in function, while also adversely affecting social and intellectual development of children, productivity among adults and the costs of treatment.

Investment in effective clinical prevention programmes provides a potential evidence-based approach to improving the oral health of populations while avoiding the social impact of oral disease. However the prevalence of prevention in some populations is low, while in others where prevalence of prevention is greater, the distribution of preventive care may not reflect the distribution of needs for prevention in the population. This indicates that although we may have information on what works in prevention under study conditions (efficacy), this has not been matched with evidence on what is required to ensure that these preventive procedures reach the populations in need (effectiveness).

It may be that providers are unaware of the evidence on prevention and, unlike the services they provide for treatment of disease, they are unable to see the outcomes of preventive services at the level of the individual patient since they do not know when disease would have occurred in the absence of prevention. This indicates that effective dissemination programmes need to be adopted (and evaluated) to ensure that providers receive, understand, believe and intend to act on evidence of effectiveness of prevention along with feedback on the achievements of prevention among the provider's client population.

Even with effective dissemination, one potential barrier to effective prevention is the way providers are remunerated or rewarded for delivering care. Providers working under fee for service (FFS) payment methods rely on a constant flow of patients with oral disease in need of treatment in order to fulfil their workload (and hence income) expectations[3]. Reducing oral disease in the population reduces the size of the future market for treatment. Hence a provider's intention to act on evidence of effectiveness may be offset by the financial implications of turning the intention into practice. Effective prevention may therefore depend upon more prevention-friendly methods of remuneration if providers are to be rewarded appropriately for doing what the system expects them to do.

The aim of this paper is to consider whether changing the way providers are paid for delivering care can be expected to change the utilisation of preventive care in the population in terms of the proportion of the population receiving preventive care, the distribution of preventive care in the population and the pattern of preventive care received (timing and content).

### Linking provider payments to system objectives

The goals and objectives of a health care system usually reflect the social values of the population. For example in the UK, the National Health Service was introduced through legislation with the objective of ensuring that “every man, woman and child can rely on getting … the best medical and other facilities available and that their getting them shall not depend on whether they can pay for them or any other factor irrelevant to real need”,[4] while in Canada the legislation that gave rise to the universal publicly-funded Medicare programme identified the primary policy objective being “to protect, promote and restore the physical and mental wellbeing of residents of Canada and to facilitate reasonable access to health services without financial or other barriers".[5] An interesting feature of these policy objectives is the identification of the health or health care needs of the population as the central focus of policy as well as the absence of mention of health care providers. Instead health care provision is a means of pursuing the policy objectives.

Health care providers act as both the suppliers of services (aimed at protecting, promoting and restoring health) as well being influential in determining the demand for those services (through their recommendations to patients seeking changes in health status or risks to health). As a result, the quantity, type and distribution of services delivered in a population will be largely determined by the decisions of providers. But the way dental care is organised, funded and delivered will determine who attends for care. Hence efficient use of resources depends not only on what is provided to attending ‘patients’ but also who attends for care. The well-established inverse-care law[6] indicates that those at greatest risk of disease, and hence greatest need for prevention, are often the same individuals who are least likely to attend.

If resources are to be allocated efficiently (i.e., in ways that make greatest impact on population health) the incentives presented to health care providers need to be closely aligned (or compatible) with the objectives of the health care system. Yet historically, methods of provider payment have often simply been inherited from periods prior to the adoption of current policy objectives. For example in Canada, FFS has remained the most predominant form of physician payment for over a century despite policy changes throughout the second half of the 20^th^ century aimed at using health care resources in accordance with ability to benefit, as opposed to ability to pay (the economic condition for allocative efficiency) and hence maximising expected health gain from whatever resources are committed to health care (the economic condition for technical efficiency).

Methods of provider payment are ways of sending messages to providers about how you want them to behave. They generate rewards for provider behaviour and, insofar as providers respond to these messages, they determine the allocation of health care resources (what services are produced) and the distribution of health care services (who receives those services), and hence the level of provider income.

It is important to note that choosing between methods of payment is concerned only about *how* providers are paid, not *how much* they are paid. Any income level is compatible with any method of provider payment - the methods only differ in what the provider has to do in order to receive that level of income. In other words, the choice of provider payment *method* is not about reducing (or increasing for that matter) the average income of providers, or the cost of the health care system in which they work, although often policy discussions have been based on such policy goals. Moreover, individual providers have their own goals and aspirations as well as constraints on their behaviour. As a result different providers may respond differently to the same payment method.

In some cases an individual provider may be paid 'too much’ from a system perspective in the sense that increasing the rate of payment (per hour, or per service or per client) may reduce the provider's total output (and conversely reducing the rate of payment would lead to him/her providing more time/services/clients). For example increasing the fee per service (or hour or client) from $50 to $60 for a provider producing 200 services (or working 200 hours or serving 200 clients) means that production can be reduced to 180 services (or hours or clients) while increasing total income from $10,000 to $10,800. Sometimes wage rates, fee levels or capitation rates may be increased in response to perceived provider shortages only to find the problem gets worse. This is because of conflicting ‘income’ and 'substitution’ effects of changing payment rates (as the rate of pay increases the provider can now take more leisure without reducing his total income as much as before the pay rate increase) and generates what in economics is called the ‘backward bending supply curve’.

Theoretical models and conceptual frameworks can provide the bases for generating incentive systems compatible with system objectives, but only careful empirical analysis can determine whether providers (as individuals or as a whole profession) respond to those incentives and if so, whether the response is in the desired direction.

### Conceptual framework

A conceptual framework for analysing provider payment methods is presented in Table 1. This identifies three distinct payment methods, salary, fee for service and capitation - with the important difference between the methods being the basis on which provider payments are calculated. Payment by outcomes (or results) is a fourth method where payment is based on the levels of health (or health gain) of clients. For example, the physician to the Royal Court of Siam (Thailand) at the turn of the 20^th^ century was paid a fixed fee for every day the members of the royal family were well.[7] However payment by outcomes is limited by ways of measuring the provider's contribution to health outcomes, i.e., health is produced by a range of different inputs only one of which is the health care the individual receives. To implement such an approach for an entire profession would therefore provide incentives to providers to serve those groups in the population more likely to benefit from services. Overcoming such patient selection incentives requires methods for adjusting payment by outcomes for ‘patient risks’ which essentially moves us back to capitation-type principles.

**Table 1:** A conceptual framework for provider payment methods

**Method**	**Basis of payment**	**Total expenditure**	**Increase income**
Salary	Inputs (time)	E = (E/T) × (T/P) × P	Increase time
FFS	Throughputs (services)	E = (E/Q) × (Q/P) × P	Increase services
Capitation	Responsibility (clients)	E = (E/N) × (N/P) × P	Increase clients

E = Total Expenditure, P = number of providers, T = provider hours, Q = services provided, N = clients served, E/T = wage rate per hour, E/Q = average service fee, E/N = average capitation fee

Finally a mixture of the different approaches can be used (often referred to as blended payments) producing a more complex set of provider incentives. Under each approach the actual level of payment can be adjusted to reflect between-unit variations (i.e., hours, service type and patient characteristics). So, time spent in different activities can be set to reflect the intensity or complexity of different activities, or different periods of the day under salary-based systems; under FFS, fee levels can be set to reflect the intensity, complexity or time requirements of different services; under capitation, fee levels can be set to reflect the expected needs for care of different types of clients (e.g., elderly versus young, male versus female etc).

An interesting feature of the three methods is that the focus of overall health care policy, the populations being served, is present only in capitation payments where a provider is paid on the basis of the number of individuals for which he/she accepts responsibility for providing health care (and may include responsibility for services provided by referral). Accordingly, total expenditure for any mean level of capitation fee is limited by the total size (and characteristics) of the population. Under salary payments, total expenditure is limited by the total number of providers (which is independent of the size and characteristics of the population) while there is no such natural limit to expenditures for under FFS - as long as more services can be delivered, total expenditures can increase. Although expenditure caps or ‘clawbacks’ can be used to limit expenditures under FFS, these introduce a separate set of incentives also unrelated to populations and their needs for care. Hence capitation offers a clear advantage for managing total health care expenditures. But what about the incentives generated to providers?

Under salary there is no incentive to providing care efficiently (i.e., prioritising clients with greatest needs, delivering care in ways that allow more clients to be cared for, and in ways that maximise the chances of health gain). That does not mean providers will not work efficiently under salary - but they will receive no additional reward for doing so compared with colleagues who do not work efficiently.

Under FFS, there is also no reward for working efficiently because the income received relates to the services produced, no matter what types of clients receive the services, or the quality of the services provided. At the extreme, the provider could earn his/her income by seeing only one client, but providing many services. Again, it does not mean that providers will not deliver services efficiently, but they will receive no additional reward for doing so compared with colleagues who do not, for example, deliver services based on the relative needs of clients. Moreover, delivering fewer services but of a higher quality will be associated with a lower level of income.

Finally under capitation there is an incentive to deliver care in ways that allow more clients to be cared for - because this determines the provider's income. However, within any particular client load (patient population) there is no incentive to deliver more services, or to spend more time with each client because this reduces the provider's capacity to see more clients (and hence boost his/her income).

This means we cannot expect one particular policy tool (i.e., the chosen payment method) to serve as both a rewards system for providers and a performance appraisal system for funders. Instead, whatever method of payment is to be used, careful management processes and performance appraisal mechanisms will still be required in order to ensure that provider behaviour is compatible with system objectives. However, although capitation does not provide rewards for delivering more care (as distinct from taking on more clients) and, as a result produces an incentive for what is often called 'supervised neglect’ (i.e., accepting clients but not providing them with appropriate levels/types of care), the risk to a provider of not providing appropriate care is that a client may switch providers. Because payment is related to number of clients, this presents a real threat to the provider's income level. This additional incentive to satisfy clients is absent from the other methods simply because payments do not relate to client numbers/types. Under salary payments, a dissatisfied client simply reduces demands on the providers time without affecting his/her income while under FFS dissatisfied clients moving to different providers simply frees up provider time to deliver more services or more frequent services for other clients, hence replacing any lost income from dissatisfied clients.

The incentive for patient satisfaction under capitation will depend crucially on clients being able to change providers and hence trigger the rewards system capitation is meant to provide. If, for example, providers act together to minimise client movements (by say closing their patient rosters) clients will be deterred from leaving a provider's practice even where they are dissatisfied because the opportunity to find another provider has been restricted.

This conceptual framework indicates that under FFS the incentive is to deliver more service whereas under salary and capitation the incentives are to deliver fewer services. Under capitation, unlike under salary, this incentive is mitigated by the need to retain and attract clients. Because effective prevention offers a potential way of reducing service demands per client, and hence increasing a provider's capacity to serve more clients, capitation payment in principle provides explicit rewards for prevention not offered by the other payment methods. It pays to do whatever is expected to result in fewer treatment needs.

The conceptual or theoretical issues associated with different payment methods do not reflect the current context of health care systems, the way service production and delivery is organised and the way providers are paid. It is therefore important to recognise the system context - policy makers are not operating in a vacuum (or from a blank piece of paper) but are concerned with finding ways of *changing* aspects of the current system in order to achieve changes in system outcomes. As a result, we need to consider whether applying the conceptual framework (i.e., changing provider payment methods) to existing systems of care changes the balance between treatment and prevention and increases the proportion of the population receiving evidence-based dental care.

### The effects of changing payment methods

The role of dental care in publicly-funded dental care systems varies across jurisdictions. In some cases it is largely excluded from the system (e.g., The Canada Health Act is limited to services provided in hospitals and services provided by physicians thus excluding most dental care services and providers) while in others it remains subject to the same principles as medical care (e.g., UK National Health Service).

Even in systems where dental care is a formal part of the publicly funded system, in many cases services are delivered under contract by ‘private’ dentists with contracts generally based on FFS arrangements. Moreover, fiscally strained health care systems often look to increasing user payments for dental care as ways of reducing the costs of dental care to the health care system, further reducing the difference between publicly funded and privately funded dental care.

The main areas where this is not the case are in Public Dental Health clinics and services for the Armed Forces where providers are employees and paid by salary. However these form a small proportion of total service delivery and are often limited to high risk or specific need groups in populations who have problems accessing private dentists. But because of the difference in payment methods, as well as difference in the clients being served, we might expect the effect of introducing capitation payments to depend, among other things, on the prevailing form of payment and patient populations.

Brocklehurst *et al*.[8] performed a Cochrane (systematic) review of the effects of payment methods on the behaviour of primary care dentists covering randomised controlled trials (RCTs), non-randomised controlled trials (NRCTs), controlled before-after (CBA) studies and interrupted time series (ITS) studies. Out of 4737 distinct articles identified, only 13 remained after screening. Eight of these 13 articles were then excluded because of inappropriate study designs (uncontrolled before and after studies, inadequate number of time points) leaving 5 articles based on two different studies using experimental (RCT) designs.

The first study involved evaluating the effects of *changing dentist payment from FFS to capitation* for the delivery of dental care to children under the UK NHS.[9] The study compared care provided to children by dentists for whom payment *changed* from FFS to capitation fees (the intervention group) with to care provided to children by dentists who had no change in payment method (continued to be paid by FFS) (the control group). Dentists in the intervention group were paid FFS for care required at the start of the intervention to render each child dentally fit before payment was then switched to capitation for future care. The intervention lasted for three years.

The second study involved evaluating the effects of *changing payment from capitation to capitation plus targeted FFS*(i.e., blended payments) for specified preventive procedures for the delivery of dental care to children in Scotland under the UK NHS.[10] The study compared care provided to children by dentists paid by capitation, but in the case of one group, an additional incentive of FFS payments for targeted preventive care was introduced.

### Changing from FFS to capitation

This study involved four paired comparisons of dental care of children based on selected UK communities (rural, urban, suburban and Scotland, which has markedly poorer oral health than the other parts of the UK).[9] The mean number of filled teeth per child and the mean percentage of children having one or more teeth extracted were lower among children living in communities served by dentists paid capitation compared to children from communities served by dentists paid FFS. However the mean percentage of children receiving active preventive advice was higher in communities served by dentists paid capitation. The prevalence of decayed teeth was higher in communities served by dentists paid capitation but this difference was only statistically significant in one of the pairs of communities. Brocklehurst *et al*.[8] report that “dentists working under capitation arrangements restored carious teeth at a later stage in the disease process than those working under fee-for-service arrangements, but this delay did not appear to compromise dental health.". Although not all of the paired comparisons were statistically significant, the authors of the review noted that the unit of analysis (e.g. dentists, patients) was often not the same as the unit of randomisation (communities), leading to unit-of-analysis error, while the baseline mean decayed/missing/filled permanent teeth (DMFT) were unbalanced in two of the four pairs, with the differences favouring the FFS communities.

### Changing from capitation to capitation plus targeted FFS

The mean percentage of 12-14 years olds receiving fissure sealants on second permanent molars was 7.1% higher among those served by dentists receiving the additional FFS payment than among those served by dentists who received only capitation.[10] This difference was not statistically significant. However after adjusting for the deprivation category for the area of practice, the number of partners in the practice, the throughput of 11 to 13-year-old patients, and the number of restorative fissure sealants placed on first permanent molars at baseline, the percentage difference in children receiving the sealants between those served by dentists with the FFS incentive and those served by capitation-only dentists increased to 9.8% and was statistically significant (p<0.05), albeit with a very wide 95% confidence interval (1.8% to 17.8%). The authors of the review noted that the study results need “to be interpreted in the context of a high risk of bias and indirectness” and questioned the policy significance of an intervention where a 1.8% improvement fell within the range of statistical significance.[8]

One point not discussed in the review was the absence of control on the total costs of paying dentists for caring for the children. Dentists receiving additional FFS for targeted services provided more care for which they received more pay. However the study did not consider how dentists would behave without the additional FFS for targeted services but with enhanced capitation levels. In other words the study involved comparing not only different *forms* of remuneration but also different *levels* of remuneration. As a result the findings cannot be interpreted as evidence of the effects of the blended payment compared to capitation. The authors went on to calculate a cost-effectiveness ratio which is only relevant where total costs of the alternative options are different. But they provide no rationale for considering only one way of using these additional resources to improve preventive practice.

### Non-experimental study designs - differences between providers under different payment methods

Several other studies have analysed *differences* in service use, mix, frequency and outcomes associated with different provider payment methods using non experimental designs. These studies represent a range of policy comparisons but are not designed in ways that inform policy makers about the effect of *changing* payment method on provider behaviour. For example Chalkley *et al*.[11] found that treatment intensity was greater where dentists were paid capitation compared to dentists paid salary in the UK NHS. However there were significant differences in the types of patients being served by the two groups as well as provider self selection into type of payment method. Atchison and Schoen[12] found that in the US, patients of dentists paid by FFS had more visits and services than patients of dentists paid by capitation. Overtreatment, occurred among dentists paid by FFS practices and under-treatment occurred among dentists paid by capitation where over and under treatment were measured by comparison of the prevalence of ‘complex’ treatments with explicit guidelines for those treatments. Grytten[13,14] compared behaviour of dentists under a salary plus capitation blended payment method with behaviour of dentists under a regular salary payment method in Norway. Providers self selected into the blended payment method. No differences were found in the levels of prevention delivered, or under-diagnosis of treatment needs and under-treatment of diagnosed conditions. Providers under blended payment methods did not select among patients but they did take responsibility for more patients.

In the UK NHS provider payment arrangements changed in 2006 with a FFS system being replaced by three ‘course of treatment’ payment bands. As a result many items of treatment that previously carried different fee levels were now paid the same amount. An analysis of trends in service items found that within a very short period under the new payment arrangements the mix of service types changed rapidly with services that require less dentist time (e.g., extractions) ‘replacing’ services that require more dentist time (e.g., restorations).[15]

Whittaker and Birch[16] found that the same change in payment methods led to some dentists self selecting ‘out’ of the NHS (i.e., a reduction in total NHS supply) and a redistribution of care provided under the NHS away from clients with regular visits and towards ‘hard to reach’ client populations. However the payment change was accompanied with the adoption of ‘commissioning’ of NHS dental care with local health trusts now contracting for the delivery of NHS care. Methodologically these two policy changes cannot be separated.

## Summary

There is limited evidence available on the effects of changing payment methods on provider behaviour. The recent Cochrane review [8] found only two studies using experimental designs to evaluate provider responses to change in remuneration method. However even in these studies the risk of bias was judged to have been high and the quality of the evidence generated, using the GRADE system[17], was assessed as low or very low.

Notwithstanding the limitations on the volume and quality of evidence, it appears that one common finding from these studies is that providers do respond to financial incentives, although the nature of that response encompasses a wide range of possibilities including the selection of patients being cared for, the self-selection of providers into preferred payment methods (including in the case of the 2006 NHS reforms, providers withdrawing from the publicly funded system) and the behaviour of providers following change in payment methods. Moreover the direction and quantity of change is difficult to predict. It may depend on the context in which the change is occurring. For example, introducing capitation as a method of provider payment may have a different effect on providers who were previously paid salary to the effect it has on providers previously paid by FFS. Even within groups of providers with the same existing payment method we might expect variations in the effects of changing payment methods among providers because they differ in their preferences for leisure and income (and hence the value to them of trading off one for the other) as well as the level and distribution of needs in their patient populations.

Capitation payments support prevention and reward providers for delivering preventive programmes by linking provider income to the volume and type of clients for whom the provider takes responsibility. Prevention among high risk clients can be prioritised by setting capitation fees in accordance with policy priorities in order to reward providers for adopting strategies for outreach among ‘hard to reach’ populations and retention of new or irregular attending clients. Whether providers respond to these incentives, and hence take advantage of the additional income opportunities, is an empirical matter which is likely to depend on the design of the capitation system (e.g., the levels of capitation fees, variation in those fees by client type, provisions for supporting excessive and unanticipated client needs etc).

Beyond these incentives for prevention, capitation has deterrents to under-treatment where clients have the capacity to move between providers and provides a way of managing the total cost of dental care by linking expenditures to the needs of the population as opposed to the size or characteristics of the provider workforce independent of those needs. However patient satisfaction may not be closely correlated with quality of care indicating that a more informed patient population might be required in order to effectively reduce incentives to ‘underserve’. Based on the range of studies reviewed, capitation has been associated with providers taking on more clients, delivering more prevention among clients and delaying the point of intervention in the disease processes.

Policy discussions aimed at introducing, promoting or expanding prevention through, among other things, provider payment reform need to carefully evaluate the response of providers to such reforms.

## Competing interests

The author received funding to attend the Cape Town Conference from Colgate Palmolive and declares no other competing interests.

## Author's information

SB is professor of health economics at McMaster University and University of Manchester. He has published widely on the economics of dental care, served as visiting professor at dental schools in Canada and Sweden, collaborated with dental researchers and served on task forces, working groups and expert panels in many countries.

## Declarations

This article has been published as part of *BMC Health Services Research* Volume 15 Supplement 1, 2015: Proceedings of Prevention in practice - making it happen. The full contents of the supplement are available online at http://www.biomedcentral.com/bmchealthservres/supplements/15/S1. Publication charges for this supplement were funded by Colgate Palmolive.
